# Acute Dystonia Due to Citalopram Treatment: A Case Series

**DOI:** 10.5539/gjhs.v6n6p295

**Published:** 2014-10-28

**Authors:** S. Mohammad Moosavi, Mahshid Ahmadi, Mani B. Monajemi

**Affiliations:** 1 Department of Psychiatry, Mazandaran University of Medical Sciences, Psychiatry and Behavioral Science Research Center, Sari, Iran; 2 Department of Clinical Psychology, Tehran University of Medical Sciences, Tehran, Iran

**Keywords:** acute dystonia, citalopram, case series

## Abstract

**Introduction:**

Abnormal movements such as acute dystonia, dyskinesia, parkinsonism, exacerbation of Parkinson disease, akathisia and possibly neuroleptic malignant syndrome may be associated with the use of selective serotonin reuptake inhibitors (SSRIs) rarely. Citalopram, a typical SSRI, used in serotonergic dysfunction related disorders, potentially can cause extrapyramidal symptoms such as acute dystonia.

**Methods:**

In a retrospective survey on patients referred to psychiatric clinic between February 2010 and February 2011 who were prescribed citalopram by the psychiatrist. The data about Demographic, history of drug and alcohol abuse or dependence, diagnosis and citalopram consumption length collected. The daily dose of citalopram was also recorded. Acute dystonia was identified by a validated chart review and precise neurological examination.

**Results:**

Nine patients were included. Citalopram was initiated at a 20 mg and titrated to a mean dose of 27 mg. The median length of acute dystonia after citalopram therapy was nine days. Other common adverse events included somnolence, gastric upset and nightmare in the cases.

**Conclusions:**

This case series was an effort to show the citalopram potential to trigger acute dystonia. Clinician needs to be aware of possible dystonia, as early recognition is necessary to prevent major adverse outcomes.

## 1. Introduction

Dystonia is a syndrome of involuntary, repetitive (or sustained) muscle contractions of opposing muscles, which may result in torsions and abnormal postures (Seeman et al., 2008). Dystonia are a clinically and genetically heterogeneous group of movement disorders. Dystonia can be the only sign of the disease or maybe only one of several manifestations of clinical syndrome (Klein & Münchau, 2013) such as Acquired brain lesions, degenerative disorders, or may be drug-induced or even psychogenic. Primary dystonia is believed to be very rare but may be underestimated (Wichowicz et al., 2009). Drug-induced dystonia may occur within minutes or hours or even days of exposure to an inciting drug; it may be observed with familial pattern and can or cannot be correlated with blood level of the drug (Mezaki, 2012). Antidepressant induced extrapyramidal symptoms (EPS) represent an under recognized but important clinical entity. These symptoms reported for duloxetine, nefazodone, bupropione and citalopram. EPS seems not dose related and can develop with short-term and long-term use (Madhusoodanan et al., 2010). Selective serotonin reuptake inhibitors (SSRIs) induced movement disorder reported in adolescents and adults (Najjar et al., 2004). Citalopram is a typical SSRI, used in serotonergic dysfunction related disorders, including depression, anxiety, panic disorder and obsessive-compulsive disorder. Besides headache, tremor is considered as a second most common neurological adverse effect of SSRIs based on literatures. Abnormal movements such as acute dystonia, dyskinesia, parkinsonism, exacerbation of parkinson disease, akathisia and possibly neuroleptic malignant syndrome may associated with the use of SSRIs very rarely. There is citalopram-induced bruxism, serotonin syndrome and jaw tremor case reported in literatures (Celik & Balci, 2010; Kinling et al., 2010). In view of the risk of morbidity and decreased quality of life and/or even mortality in case of laryngospasm due to contraction of laryngeal muscles contraction laryngeal dystonia is a life-threatening side-effect of and its diagnosis often remains elusive (Christodoulou & Kalaitzi, 2005) and to aware the clinicians of potential to cause adverse effects; we reported the nine cases developed acute dystonia following administration of citalopram, as a very rare condition.

## 2. Materials and Methods

This case series was a retrospective study and carried out in a psychiatric clinic in Sari (Iran) that has more than 5000 patients annually. This review was unfunded and we reviewed the case files from February 2010 to February 2011 who were under citalopram treatment by the psychiatrist for variety of depressive disorder and anxiety disorders (Obsessive-compulsive disorder, General anxiety disorder, Panic disorder and Posttraumatic stress disorder). Before initiation of citalopram prescription, all antidepressants that the patients may have been taking were discontinued, and Patients were excluded if there were taking any psychiatric drugs (except benzodiazepines). The data about demographic, diagnosis, drug or alcohol dependence and abuse (based on DSM-4-R), and length of citalopram consumption, citalopram dosage were gathered. The initiation daily dose of citalopram dose and dose changes were and also adverse effects (symptoms and signs) at the time of citalopram therapy recorded. Beside clinical examination, a retrospective chart review was performed to identify dystonia. Brain MRI, Physical examination, and neurological examination were performed in the cases of acute dystonia. Laboratory tests such as CBC, ESR, BS, LFT, CPK, TFT was also done in the manner.

## 3. Results

A total of nine patients were diagnosed with acute dystonia, none of them were excluded by the exclusion criteria, hence; all of them were included in the case series (Table 1). Three Patients were male and six were female with the mean age of 29 years. Drugs and alcohol abuse and dependency recently had not seen in any of patient’s notes (according to DSM-4-R criteria). Citalopram was initiated at 20 mg per day prescribed in single dose and titrated by the psychiatrist for all patients according to the response. The median dose administered was 27 mg per day totally (range 20-50 mg). Other side effects were recorded in all patient notes. One patient had drowsiness, short term (less than 2-3 days) mild gastric upset in three and nightmare in two, but these adverse events were transient and not prominent enough to discontinuation of citalopram. There was consistent documentation of dystonia identified in the chart review. The onset of dystonia was days 9 from initiation (Table 1). Brain MRI showed no significant pathological changes in the cases. Lab data was in normal range (except non significant CPK raise in two patients and LFT raise in two other). No pathological finding in physical examination was found. The attending clinician diagnosed acute dystonia and another clinician to verify the diagnosis did re-exam. Biperidine 5 mg administered intramuscularly for all patients after diagnosis. Full recovery was noted in four cases after the first injection and in five cases after the second administration 4 hours later. None of the cases needed further therapeutic drugs for dystonia. Citalopram changed to other antidepressants and/or antianxiety agents.

**Table 1:** Patient Characteristics

Patient Characteristics		Data
Number of patient under citalopram treatment		1875
Number of women (total)		1145
Number of men (total)		730
Number of patients with dystonia		9
Mean age		29 (24 to 41)
Males, n (%)		3 (33.3%)
Female, n (%)		6 (66.6%)
Mean days of citalopram consumption, days (range)		9 (6 to 17)
		
Diagnosis, n (%)	Anxiety disorder	3 (33.3%)
	Depressive disorder	4 (44.4%)
	Mixed	2 (22.2%)
		
Minimum initial dosage (mg)		10
Median dose of citalopram, mg (range)		27 (20- 40)
Substance abuse or dependency, n (%)		None (0%)
Alcohol excess, n (%)		None (0%)

## 4. Discussion

This retrospective study found a real association between the citalopram use as an antidepressant or antianxiety agent and acute dystonia as an adverse effect; however it does encourage the development of randomized, controlled trials of citalopram in this patient’s population. Basically drug induced acute dystonia is an uncommon adverse event; lower than 9 per 100,000 patients who receive psychotropic drugs diagnosed with this condition (Stubner et al., 2004). On the basis of this fact that antipsychotic drugs are the major and the most common causes of extrapyramidal adverse effects (Guitton et al., 2011). As aforementioned, Citalopram induced acute dystonia is a really rare event, so dystonia due to citalopram use have been remained in case reports limit. On the basis of this fact that antipsychotic drugs are the major and the most common causes of extrapyramidal adverse effect (Guitton et al., 2011). Abovementioned illustrated that Citalopram induced acute dystonia is a really rare event, so dystonia due to citalopram use have been remained in case reports limit. In this study we found 9 per 1875 (48 per 10000), which is more than other studies. The differences may be related to genetically differences between patients’ difference in nationality and races. Another potential reason may be due to different initiation dosage of citalopram, which in this survey was 10 mg just for 1-3 days and titrated after these days. The other reason can be the diagnosis. The diagnosis of dystonia may tend to be delayed after the onset of symptoms, or the symptoms may be left unrecognized or misinterpreted. “Dry eye” is common in the modern society and is a misdiagnosis of blepharospasm. “Stiff sensation of the neck”, an usual complain in some type of jobs (musicians, golfers, typists and etc.…) may actually be a phenotype of cervical dystonia so dystonia is common against a general belief, and should be included `among the differential diagnosis in patients with muscular hyperactivity and impaired voluntary movements (Mezaki, 2012). Although motor disorders are well reported with SSRIs, there have been few reports with Citalopram being thought to have very low potential for extrapyramidal side effects. This sort of side effects is thought secondary to an indirect modulatory effect of dopaminergic function through inhibitory serotonergic input (Thwaites, 2006). Among extrapyramidal effects, acute dystonia, which is a painful contraction of a group of muscles, occurs because a relative imbalance between decreased dopamine and increased Acetylcholine activity in basal ganglia. Citalopram treatment can diminish dopamine turnover as a result of increased serotonergic activity in the inhibitory raphe nucleus projections to nigral cells (Najjar & Price, 2004; Huot et al., 2011). Citalopram as a potent and the most selective SSRI can inhibit dopamine cell and synaptic release and probably synthesis of dopamine in the midbrain, striatum and cortex (Celik & Balci, 2010). The other mechanism for EPS with SSRI maybe the decrease thyrosine hydroxylase positive cell count, alter the regulation of thyrosine hydroxylase and induces microgelial activation in the substantia nigra (Sckand et al., 2011). Citalopram can induce parkinsonian syndrome (Hedenmalm et al., 2006; Kelan & Ellingord, 2009), head tremor (Celik & Balci, 2010), dyskinasia (Muthusami et al., 2009) persistent EPS (Bilen et al., 2008) may be with the same mechanism. Even delirium reported with Citalopram (Stubner et al., 2004). Above-mentioned mechanism probably explains the etiology of dystonia due to Citalopram. These explanations may clear that why no extrapyramidal effects were observed in any patients in the first couple of days. These changes such as inhibition of serotonergic input which makes dopaminergic function modulation in raphe nucleus, decreased dopamine and increased acetylcholine in basal ganglia, decrease of dopamine synthesis in midbrain, induce microglial activation need time. Some of these changes are related to neurotransmission, but others to cellular changes and the last types need more time to complete. Although there was not cleared that how much time cells need to change like this but it may explain the delayed onset of extrapyramidal side effects. This explanation probably cannot clarify much-delayed onset of dystonia (even month) reported by a few of case report study. More investigation is needed. The Risk of EPS with SSRIs may increase with advanced age. The other possible risk factor is the presence of A1 allele of d2 dopaminergic receptor gene polymorphism (Bilen et al., 2008; Parvin & Swartz, 2005). These risk factors have not described well yet. We also need further investigation to clear why some patients have EPS and others have not and why this condition occurred three month after administration in our case instead of earlier, as expected based on other reports (Najjar & Price, 2004; Seeman et al., 2008). It is obvious that the higher dose of citalopram can cause the higher risk of adverse effects and this may related to dystonia. In patients taking high dose citalopram, marked reductions in the prescribed dose of other interacted drugs can be advised. Another fact is that Dystonia can also be induced by compounds other than citalopram such as antipsychotics, especially high potency antipsychotics and even a second-generation Antipsychotics, antidepressants, levodopa, carbamazepine, dextroamphetamine, and diphenylhydantoin (Seeman et al., 2008). In these cases, it may be transient and generally disappearing after the dose is reduced or the causative drug is stopped but prescribers should be vigilant for synergistic effects of drug induced dystonia. None of patient has history of alcohol/substance abuse or alcohol/substance dependency in the last year so the effect of alcohol/substance on extrapyramidal adverse effect have not surveyed in this study. These drugs can change hepatic metabolism process so can interact on citalopram metabolism potentially. On the other view, have effects on secondary messengers, a variety of neurotransmitters and transient or permanent cellular changes in CNS. All of these changes may make the patients vulnerable to extrapyramidal side effects. Six of patients with acute dystonia (66.6%) were female. The difference was not statistically meaningful (5 per 1000 in women/4 per 1000 in men). This result is against the expectation. Nearly all documented data in papers and textbooks have been showed that acute dystonia is more in men than women. The difference may be related to this fact that women are more sensitive to their signs and symptoms psychologically so, they may consider to their body changes more. The other reason is total women under treatment is more than men there. Therefore the difference was made (1145 female/730 male). The majority of acute dystonia reported due to antipsychotics and the perhaps dystonia due to citalopram has a different epidemiology, of course this concept is hypothesis besides the view of the same pathophysiology of dystonia with any causes and needs to prove. Although some reports have been shown that SSRI induced EPS can be a dose independent side effect, but documents are very few and this hypothesis should prove with large clinical trials. Of course this type of study will be difficult in this field because of low incidence rate. There are very few reports on acute dystonia due to Citalopram, but clinician needs to be aware of possible dystonia, as early recognition is necessary to prevent major adverse outcomes.
